# Supplementary material for: The outer kinetochore components KNL-1 and Ndc80 complex regulate axon and neuronal cell body positioning in the C. elegans nervous system
Source: Mol Biol Cell. 2024 May 20;35(6):ar83. doi: 10.1091/mbc.E23-08-0325 (PMC11238089; doi:10.1091/mbc.E23-08-0325)
Supplement: Supplementary file 1 [file mbc-35-ar83-s001.pdf]

Supplemental Materials

Molecular Biology of the Cell

Ouzounidis *et al*.

Supplemental information

The Outer Kinetochore Proteins KNL-1 and Ndc80 complex are Required to Pattern the Central Nervous System

Vasileios R. Ouzounidis[1], Mattie Green[1], Charlotte de Ceuninck van Capelle[1], Clara Gebhardt[1], Helena Crellin[1], Cameron Finlayson[1], Bram Prevo[1] & Dhanya K. Cheerambathur

Figure S1

Ouzounidis et al.

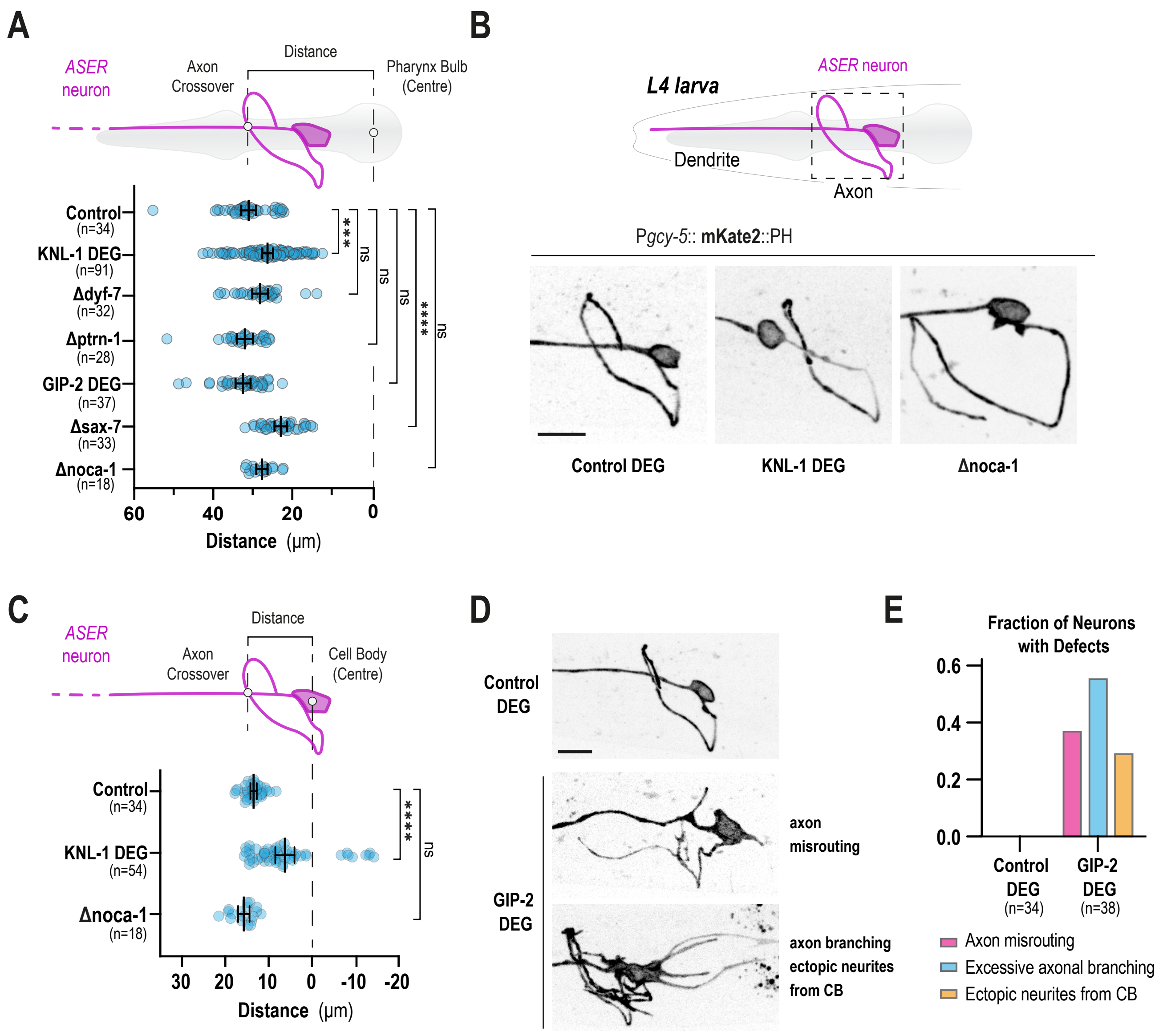

Figure S1

(A) Cartoon representation of the ASER neuron relatively to the terminal pharyngeal bulb of an L4 larva (top). Plot of distance between the pharynx bulb and the axon-dendrite intersect in the indicated conditions. n represents the number of animals. ***, **** and ns indicate $p<0.001$, $p<0.0001$ and non-significant, respectively. (B) Cartoon representation of the ASER neuron in an L4 larva (top). Crop-out images of the ASER axon and cell body in control, KNL-1 DEG and Δnoca-1. Scale bar, 10μm. (C) Illustrated schematic of the ASER neuron (top). Plot of distance between the centre of the ASER cell body and the axon-dendrite intersect in the indicated conditions (bottom). Control and KNL-1 DEG data are the same as in Figure 1D. n represents the number of animals. (D) Images of the range of ASER axonal phenotypes resulting from the post-mitotic degradation of GIP-2 using *dyf-7* driven GFP nanobody degrader system. GIP-2 degradation led to axon misrouting, where the axon did not follow the typical nerve ring trajectory, ectopic neurite formation, sprouting from the ASER cell body (CB), and ectopic axon branching. Scale bar, 10μm. (E) Quantification of the ASER axonal phenotypes of the control and GIP-2 DEG animals. n represents the number of neurons.

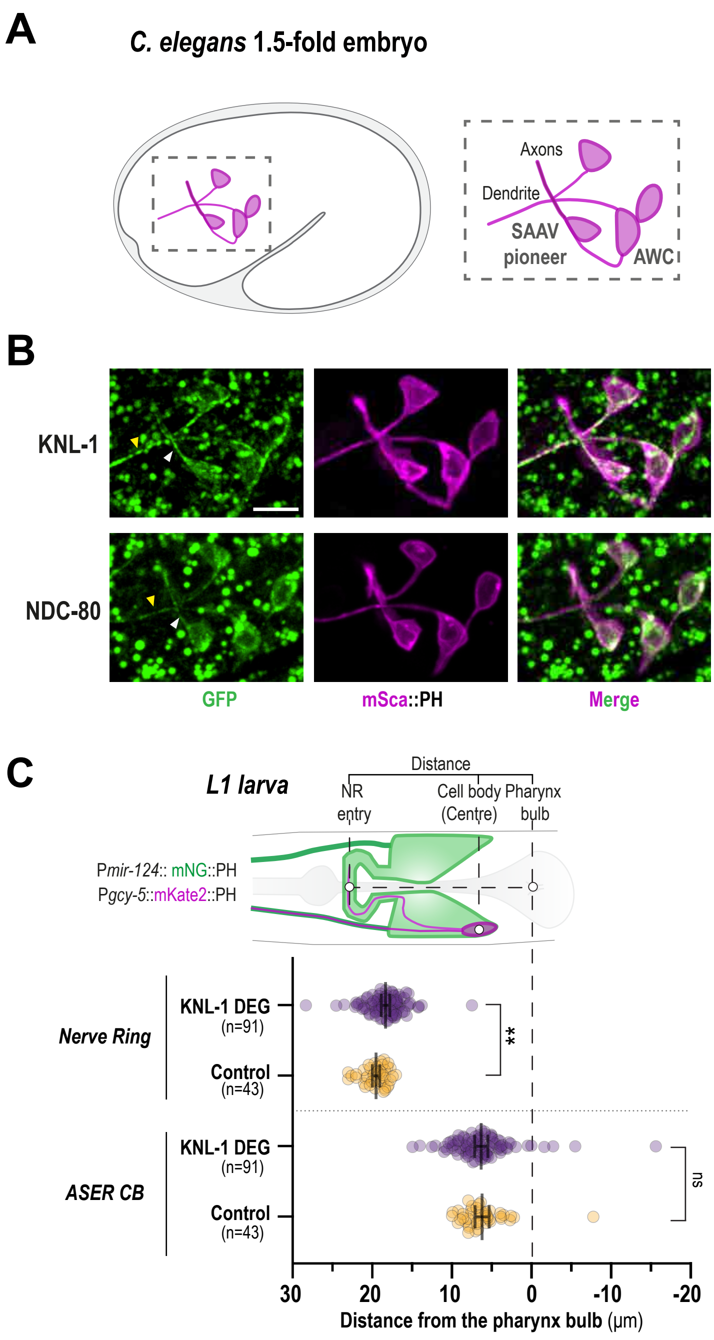
A
C. elegans 1.5-fold embryo
Axons
Dendrite
SAAV pioneer
AWC
B
KNL-1
NDC-80
GFP
mSca::PH
Merge
C
L1 larva
Distance
NR entry
Cell body (Centre)
Pharynx bulb
Pmir-124:: mNG::PH
Pgcy-5::mKate2::PH
Nerve Ring
KNL-1 DEG (n=91)
Control (n=43)
**
ASER CB
KNL-1 DEG (n=91)
Control (n=43)
ns
30
20
10
0
-10
-20
Distance from the pharynx bulb (µm)

Figure S2

(A) Schematic illustration of a lateral view of a 1.5-fold C. elegans embryo highlighting P*hlh-16* expressing neurons. *hlh-16* is active in several neurons, including the AWC amphid neuron and the SAAV pioneer neuron, from bean stage of embryogenesis until L1 larva stage. The SAAV pioneer axon that forms the scaffold of the nerve ring has extended and integrated into the nerve ring structure. (B) Localization of GFP::KNL-1 and NDC-80::GFP in developing embryonic neurons, using a split-GFP system. Neuronal membranes are labelled with mSca-I::PH (magenta). White arrowheads indicate the presence of KNL-1 and NDC-80 in the axon of SAAV. Yellow arrowheads point out the presence of KNL-1 and NDC-80 in the AWC dendrite. Scale bar, 5μm. (C) Schematic illustrating the positions of ASER (magenta) and the P*mir-124* expressing sensory neurons (green) (top). Two different measurements were quantified from the images: distance of the pharynx bulb from the nerve ring (middle) and from the ASER cell body (bottom). The distances were measured as represented in the schematic. Compared to the control, the ASER cell body maintained the same position, while the nerve ring was misplaced anteriorly, relatively to the pharynx bulb, in KNL-1 DEG animals. n indicates the number of animals. Error bars denote 95% confidence interval. ** and ns indicate $p<0.01$ and non-significant, respectively.

Figure S3

Ouzounidis et al.

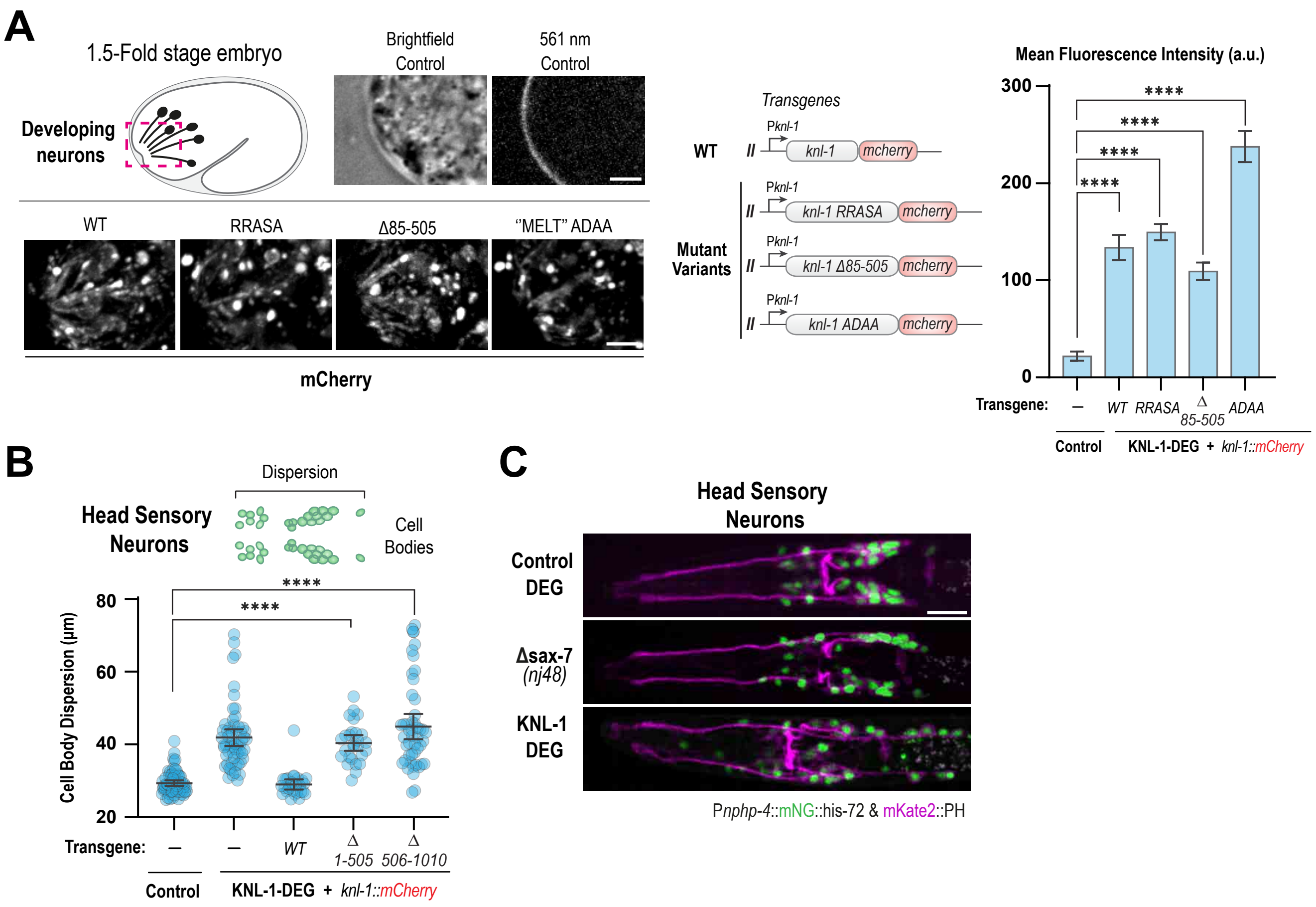

Figure S3

(A) Localization of KNL-1 variants in the developing dendrites of the sensory neurons in the 1.5-fold stage of embryogenesis, using the gene replacement system. Cartoon representing the developing dendrites in the embryo (top-left). The images at the top show a region of the embryo head of the control using Brightfield and fluorescence at 561nm. In the control there is no KNL-1::mCherry enriched in the dendrites. In the full length KNL-1 rescue (WT) KNL-1 is found in the dendrites. Similar localization pattern was observed in the expression of the KNL-1 mutant alleles (RRASA, Δ85-505, ''MELT'' ADAA) in the background of the KNL-1 GFP degrader. Scale bar, 3μm. The schematic in the middle illustrates the various KNL-1 transgenes used in this assay. The different transgenes are expressed under *knl-1* promoter and they are fused to mCherry. The graph on the right represents the quantification of the mean fluorescence intensity of KNL-1::mCherry in the dendrites in the indicated conditions. Error bars denote 95% confidence interval. **** indicates $p<0.0001$. (B) Quantification of sensory neuron cell body dispersion in the indicated conditions. The gene replacement system was used to express two alleles of KNL-1: one lacking the N-terminal signalling hub (Δ1-505), the other lacking the C terminal Ndc-80 complex recruitment module (Δ506-1010). Deletion of either region significantly impacted the dispersion of head sensory neuron cell bodies. The effect of C-terminal deletion appears more pronounced based on the distribution of the values. n represents the number of animals. Control and KNL-1 WT and DEG data are the same as in Figure 3E. **** indicates $p<0.0001$. (C) Images of head sensory neuron nuclei (green) and plasma membranes (magenta) for the indicated conditions. Images of Control and KNL-1 DEG are same as Figure 3D. Scale bar,10 μ.

Figure S4
Ouzounidis et al.

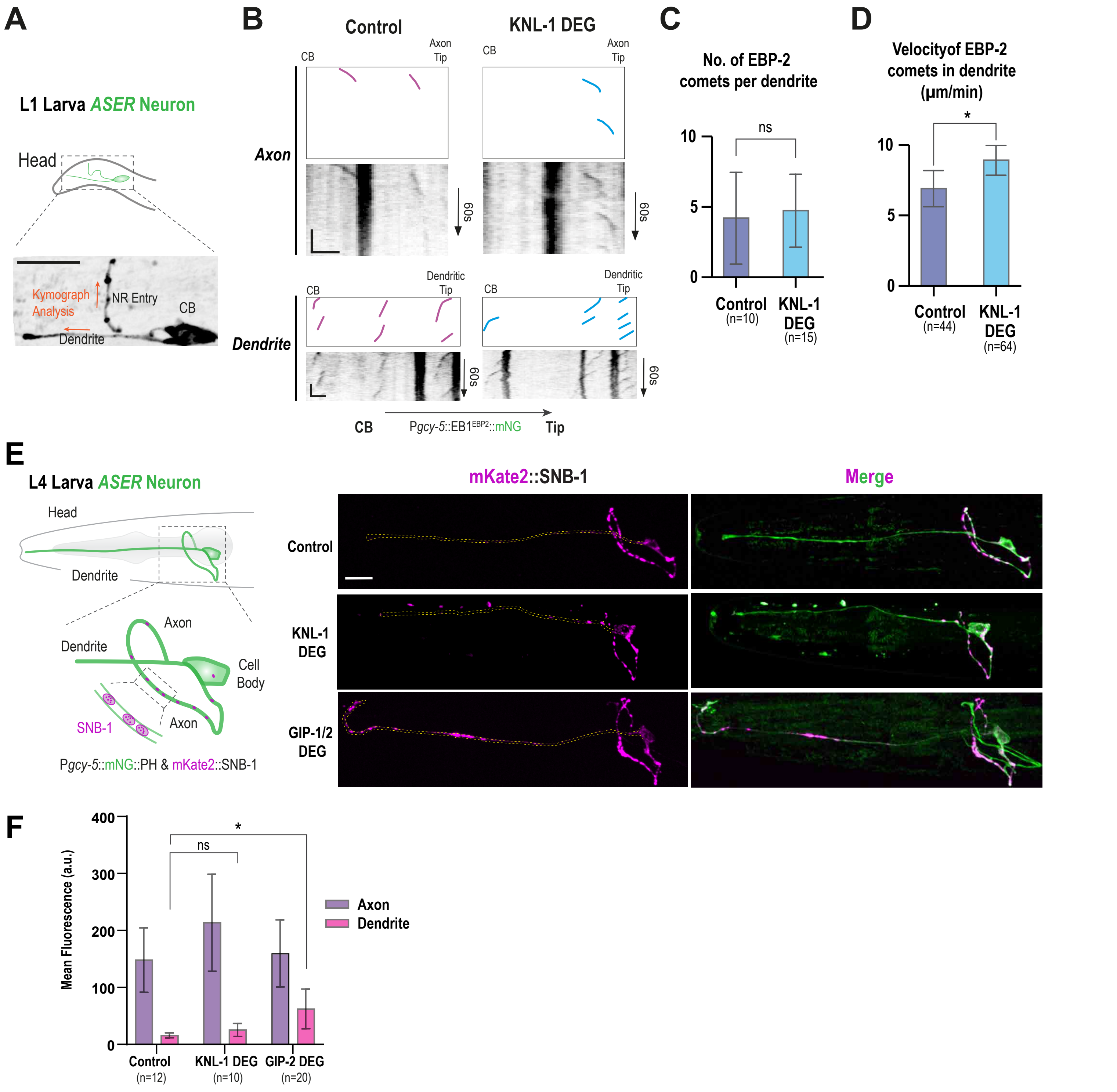

Figure S4

(A) $EB1^{EBP-2}$::mNG dynamics in the ASER axon and dendrite. The image is a maximum intensity projection of a single time frame of $EB1^{EBP-2}$::mNG movie. Scale bar, 10µm. (B) Kymographs of $EB1^{EBP-2}$::mNG puncta in axons and dendrites of control and KNL-1 DEG animals. Axon kymographs were generated for the last section of the axon that enters the nerve ring, while for the dendrite, the kymographs were generated from the whole dendritic region. The direction of the kymograph is indicated by the orange arrow in (A). Scale bar, 2µm (horizontal), 15s (vertical). (C-D) Quantification of the number and velocity of comets per dendrite. Error bars denote 95% confidence interval. n represents the number of dendrites. * and ns indicate $p<0.05$ and non-significant, respectively. (E) Schematic showing the localization of synaptic marker SNB-1 in the ASER axon. To visualize ASER neuron morphology and SNB-1 protein localization, the transgenes P*gcy-5*::mNeonGreen and P*gcy 5*::mKate2::SNB-1 were utilized, respectively. The images on the right depict the localization of synaptic marker SNB-1 (magenta) in the axon and dendrite (highlighted with yellow dashed line) of the ASER (green) in control, KNL-1 DEG and GIP-2 DEG. SNB-1 puncta are found ectopically in the dendrite of the ASER post GIP-1/2 degradation, and not in the dendrites of control and KNL-1 DEG. Scale bar,10 µm. (E) Quantification of SNB-1 signal in the axon and dendrites, in the indicated conditions. Control and KNL-1 DEG data are the same as in Figure 4G. Error bars denote 95% confidence interval. n represents the number of animals. * and ns indicate $p<0.05$ and non-significant, respectively.

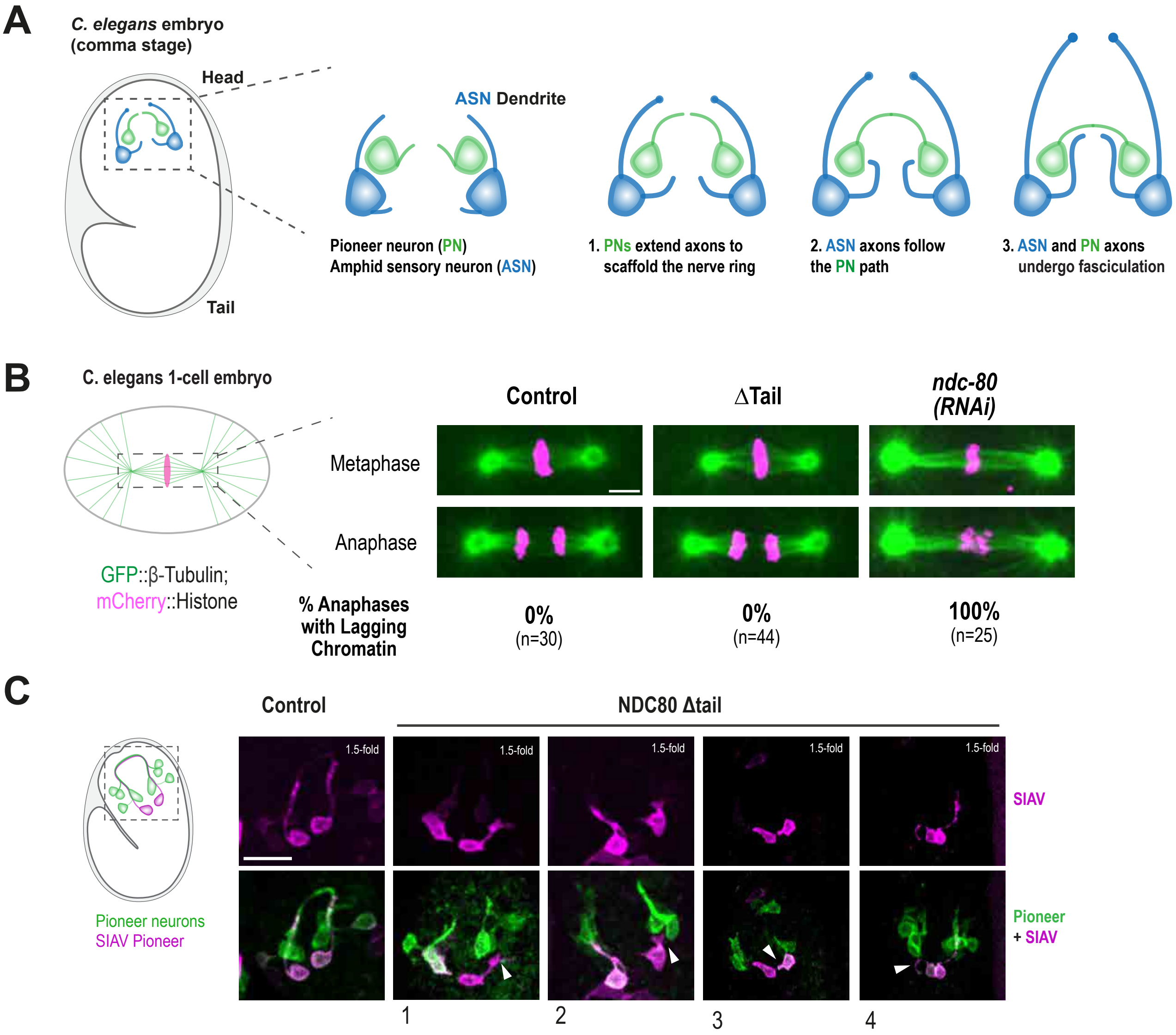
A
C. elegans embryo
(comma stage)
Head
Tail
ASN Dendrite
Pioneer neuron (PN)
Amphid sensory neuron (ASN)
1. PNs extend axons to scaffold the nerve ring
2. ASN axons follow the PN path
3. ASN and PN axons undergo fasciculation
B
C. elegans 1-cell embryo
GFP::β-Tubulin;
mCherry::Histone
Control
ΔTail
ndc-80 (RNAi)
Metaphase
Anaphase
% Anaphases with Lagging Chromatin
0%
(n=30)
0%
(n=44)
100%
(n=25)
C
Control
NDC80 Δtail
1.5-fold
SIAV
Pioneer + SIAV
Pioneer neurons
SIAV Pioneer
1
2
3
4

Figure S5

(A) Schematic of the nerve ring assembly in a C. elegans embryo. At comma stage, pioneer neuron axons extend to create the ring-shaped nerve ring scaffold. Subsequently, the axons of follower neurons, including the amphid sensory neurons, track the pioneer axons and undergo fasciculation to form the densely interconnected axonal structure in the nerve ring (Rapti et al. 2017; Moyle et al. 2021). (B) Schematic illustration of single-cell *C. elegans* embryo undergoing mitotic metaphase (left). Microtubules are labelled using GFP::TBB-2 (green) and the chromosomes are labelled using mCherry::H2B (magenta). Images on the right show the spindle and chromosomes during metaphase and anaphase of mitosis in the indicated conditions. The percentage of anaphases with lagging chromosomes is shown at the bottom of each panel. Upon NDC-80 depletion from the embryos through dsRNA mediated silencing, the chromosomes fail to align properly, resulting in lagging chromosomes during anaphase. In contrast, the NDC 80 ΔTail deletion did not show alignment defects or chromosome missegregation, suggesting that chromosome segregation was occurring normally in this condition. Scale bar, 5μm. (C) Schematic representation of the lateral view of a *C. elegans* embryo at 1.5-fold stage highlighting the pioneer neurons that form the nerve ring scaffold (left). Pioneer neurons were labelled using P*lim-4*::mNG::PH; P*ceh-17*::mSca::PH expression was restricted to SIAV neuron. Such labelling enabled tracking of the entry of SIAV axon into the pioneer axon bundle. The images display proper nerve ring assembly in the control 1.5- fold embryo alongside examples of SIAV (magenta) of axon bundling failures and inability to incorporate into the nerve ring scaffold (green). Examples 1,2 and 4 show cases where the SIAV axons are initially guided correctly towards the nerve ring but subsequently fail to extend (white arrowheads). In example 3 the SIAV axons fail to extend. Scale bar, 10μm.

Supplementary methods

RNA-mediated Interference

The RNAi against ndc-80 was performed by microinjection. Double-stranded RNAs were generated as described (Oegema et al., 2001) using DNA templates prepared by PCR-amplifying regions using the oligonucleotides:

5'-GATGACAAGTACATTCAGAGATTATACAAATGATC-3'

5'-GTGGTTCAAGATTCATTTGAATATTAAGTCCACTG-3'

and N2 genomic DNA as the template. L4 hermaphrodites were injected with dsRNA and incubated at 20°C for 36–46 h before dissection and imaging of their embryos.

Fluorescence microscopy and analysis

To visualize the expression of GFP::KNL-1 and GFP::NDC-80 **(Figure S2)** in embryonic neurons, a split gfp system linked to mScarlet::PH under the *hlh-16* promoter was utilized. Neurons were initially identified based on mScarlet::PH marker expression, and 15x0.5µm z-stacks were acquired to capture all the neurons.

<u>Table S1: *C. elegans* strains used in the study</u>

GENOTYPE	SOURCE	IDENTIFIER	FIGURE
C. elegans N2 Bristol	Caenorhabditis Genetics Center	N2	
lad-1(nj48)	<u>PMID: 15775964</u>	IK0441	**1 & 3 & S1 & S3**
ltSi220[pOD1249/pSW077; Pmex-5::GFP-tbb-2-operon-linker-mCherry-his-11; cb-unc-119(+)]I	PMID: 26371552	OD868	**S5**
ltSi1038 [pDC344; Pgcy-5::mKate-2::PH_unc-54_3'UTR; cb-unc-119(+)]II; unc-119(ed3)III	PMID: 30827898	OD3242	**1**
ltSi1016[pDC337; Pdyf-7::vhhGFP4::ZIF-1::dyf-7_3'UTR; cb-unc-119(+)]I #2; ltSi1038[pDC344; Pgcy-5::mKate-2::PH_unc-54_3'UTR; cb-unc-119(+)]II; unc-119(ed3)III; unc-119(ed3)III?; knl-1(lt53[knl-1::GFP::tev::loxP::3xFlag)III	PMID: 30827898	OD3247	**1 & S1**
ltSi1016[pDC337; Pdyf-7::vhhGFP4::ZIF-1::dyf-7_3'UTR; cb-unc-119(+)]I; ltSi1038 [pDC344; Pgcy-5::mKate-2::PH_unc-54_3'UTR; cb-unc-119(+)]II ltSi1016[pDC337; Pdyf-7::vhhGFP4::ZIF-1::dyf-7_3'UTR; cb-unc-119(+) knl-1(lt53[knl-1::GFP::tev::loxP::3xFlag])III; unc-119(ed3)III ?	PMID: 30827898	OD3249	**1 & S1**
ltSi1038 [pDC344; Pgcy-5::mKate-2::PH_unc-54_3'UTR; cb-unc-119(+)]II ; unc-119(ed3)III?; dyf-7 (it60)X	PMID: 30827898	OD3291	**1 & S1**
ltSi1016[pDC337; Pdyf-7::vhhGFP4::ZIF-1::dyf-7_3'UTR; cb-unc-119(+)]#2 I] ; ltSi1[pOD809/pJE110; Pknl-1::KNL-1reencoded::RFP; cb-unc-119(+)]II; knl-1(lt53[knl-1::GFP::tev::loxP::3xFlag]) III	PMID: 30827898	OD3346	**3 & S3**
ltSi1016[pDC337; Pdyf-7::vhhGFP4::ZIF-1::dyf-7_3'UTR; cb-unc-119(+)]#2 I] ;ltSi13[pOD858/pJE123; Pknl-1::KNL-1aa506-1010::mCherry; cb-unc-119(+)]II; knl-1(lt53[knl-1::GFP::tev::loxP::3xFlag]) III	PMID: 30827898	OD3404	**S3**
unc-119(ed3)III; ltSi1174[oxTi365; pDC591; Pnphp-4::mNeonGreen-his-72:tbb-2_3'UTR;;gpd-2/3 operon linker-mKate2-PH:unc-34_3'UTR]V	PMID: 30827898	OD3919	
ltSi1016[pDC337; Pdyf-7::vhhGFP4::ZIF-1::dyf-7_3'UTR; cb-unc-119(+)]I; unc-119(ed3)III?; ltSi1174[oxTi365; pDC591; Pnphp-4::mNeonGreen-his-72:tbb-2_3'UTR;;gpd-2/3 operon linker-mKate2-PH:unc-34_3'UTR]V	PMID: 30827898	OD3924	**3 & S3**
ltSi1016[pDC337; Pdyf-7::vhhGFP4::ZIF-1::dyf-7_3'UTR; cb-unc-119(+)]I; knl-1(lt53[knl-1::GFP::tev::loxP::3xFlag])III; ltSi1174[oxTi365; pDC591; Pnphp-4::mNeonGreen-his-72:tbb-2_3'UTR;;gpd-2/3 operon linker-mKate2-PH:unc-34_3'UTR]V	PMID: 30827898	OD3938	**3 & S3**

ltSi1016[pDC337; Pdyf-7::vhhGFP4::ZIF-1::dyf-7_3'UTR; cb-unc-119(+)]I ; ltSi120[[pDC170;Pndc-80:NDC-80 reencoded; cb-unc-119(+)]II #3; unc-119(ed3)III? ; ndc-80(lt54[ndc-80::GFP::tev::loxP::3xFlag])IV; Pnphp-4::mNeonGreen-his-72:tbb-2_3'UTR;;gpd-2/3 operon linker-mKate2-PH:unc-34_3'UTR]V	PMID: 30827898	OD3952	**3**
ltSi1016[pDC337; Pdyf-7::vhhGFP4::ZIF-1::dyf-7_3'UTR; cb-unc-119(+)]I ; ltSi121[pDC175;Pndc-80:NDC-80 (Mutant D1-59) reencoded; cb-unc-119(+)]II #1; unc-119(ed3)III? ; ndc-80(lt54[ndc-80::GFP::tev::loxP::3xFlag])IV; Pnphp-4::mNeonGreen-his-72:tbb-2_3'UTR;;gpd-2/3 operon linker-mKate2-PH:unc-34_3'UTR]V	PMID: 30827898	OD3953	**3**
ltSi1016[pDC337; Pdyf-7::vhhGFP4::ZIF-1::dyf-7_3'UTR; cb-unc-119(+)]I; ltSi711[pDC267;Pndc-80:NDC-80(66,96,100,125,144,155AAAAAA) reencoded; cb-unc-119(+)]II#1; unc-119(ed3)III? ; ndc-80(lt54[ndc-80::GFP::tev::loxP::3xFlag])IV; ltSi1055[oxTi365;[pDC344; Pnphp-4::mNeonGreen-his-72:tbb-2_3'UTR;;gpd-2/3 operon linker-mKate2-PH:unc-34_3'UTR]V	PMID: 30827898	OD3954	**3**
ltSi1016[pDC337; Pdyf-7::vhhGFP4::ZIF-1::dyf-7_3'UTR; cb-unc-119(+)] I ; ltSi1[pOD809/pJE110; Pknl-1::KNL-1reencoded::RFP; cb-unc-119(+)]II; knl-1(lt53[knl-1::GFP::tev::loxP::3xFlag]) III; ltSi1174[oxTi365; pDC591; Pnphp-4::mNeonGreen-his-72:tbb-2_3'UTR;;gpd-2/3 operon linker-mKate2-PH:unc-34_3'UTR]V	PMID: 30827898	OD3977	**3 & S3**
ltSi1016[pDC337; Pdyf-7::vhhGFP4::ZIF-1::dyf-7_3'UTR; cb-unc-119(+)]I ; unc-119(ed3)III? ; ndc-80(lt54[ndc-80::GFP::tev::loxP::3xFlag])IV; ltSi1174[oxTi365; pDC591; Pnphp-4::mNeonGreen-his-72:tbb-2_3'UTR;;gpd-2/3 operon linker-mKate2-PH:unc-34_3'UTR]V	PMID: 30827898	OD3998	**3**
ltSi1016[pDC337; Pdyf-7::vhhGFP4::ZIF-1::dyf-7_3'UTR; cb-unc-119(+)]I; ltSi1191[pDC603; Pgcy-5::mNeonGreen-PH::tbb-3'UTR; Pgcy-5::mKate2-snb-1::snb-1_3'UTR; cb-unc-119(+)] II; unc-119(ed3) III?	PMID: 30827898	OD4011	**4 & S4**
ltSi1016[pDC337; Pdyf-7::vhhGFP4::ZIF-1::dyf-7_3'UTR; cb-unc-119(+)] I] ; ltSi1191[pDC603; Pgcy-5::mNeonGreen-PH::tbb-3'UTR; Pgcy-5::mKate2-snb-1::snb-1_3'UTR; cb-unc-119(+)] II; unc-119(ed3) III; knl-1(lt53[knl-1::GFP::tev::loxP::3xFlag])III	PMID: 30827898	OD4012	**4 & S4**
ltSi1016[pDC337; Pdyf-7::vhhGFP4::ZIF-1::dyf-7_3'UTR; cb-unc-119(+)]#2 I ;ltSi13[pOD858/pJE123; Pknl-1::KNL-1aa506-	This study	DKC13	**S3**

1010::mCherry; cb-unc-119(+)]II; knl-1(lt53[knl-1::GFP::tev::loxP::3xFlag]) III; Pnphp-4::mNeonGreen-his-72:tbb-2_3'UTR;;gpd-2/3 operon linker-mKate2-PH:unc-54_3'UTR]V			
ltSi1016[pDC337; Pdyf-7::vhhGFP4::ZIF-1::dyf-7_3'UTR; cb-unc-119(+)]#2 I ;ltSi13[pOD858/pJE123; Pknl-1::KNL-1aa1-505::mCherry; cb-unc-119(+)]II; knl-1(lt53[knl-1::GFP::tev::loxP::3xFlag])III; Pnphp-4::mNeonGreen-his-72:tbb-2_3'UTR;;gpd-2/3 operon linker-mKate2-PH:unc-54_3'UTR]V	This study	DKC16	**S3**
ltSi1016[pDC337; Pdyf-7::vhhGFP4::ZIF-1::dyf-7_3'UTR; cb-unc-119(+)]#2 I; ltSi9[pOD831/pJE120; Pknl-1::KNL-1reencoded(RRASAmutant)::RFP; cb-unc-119(+)]II; knl-1(lt53[knl-1::GFP::tev::loxP::3xFlag]) III	This study	DKC67	**3 & S3**
ltSi1016[pDC337; Pdyf-7::vhhGFP4::ZIF-1::dyf-7_3'UTR; cb-unc-119(+)]#2 I; ltSi44[pOD1039/pJE170; Pknl-1::KNL-1reencoded(Mutant D85-505)::RFP; cb-unc-119(+)]II;knl-1(lt53[knl-1::GFP::tev::loxP::3xFlag])III	This study	DKC68	**3 & S3**
ltSi1016[pDC337; Pdyf-7::vhhGFP4::ZIF-1::dyf-7_3'UTR; cb-unc-119(+)]#2 I; ltSi44[pOD1039/pJE170; Pknl-1::KNL-1reencoded(Mutant D85-505)::RFP; cb-unc-119(+)]II;knl-1(lt53[knl-1::GFP::tev::loxP::3xFlag]) III;Pnphp-4::mNeonGreen-his-72:tbb-2_3'UTR;;gpd-2/3 operon linker-mKate2-PH:unc-54_3'UTR]V	This study	DKC77	**3 & S3**
ltSi1016[pDC337; Pdyf-7::vhhGFP4::ZIF-1::dyf-7_3'UTR; cb-unc-119(+)]#2 I; unc-119(ed3)III ?]; ltSi9[pOD831/pJE120; Pknl-1::KNL-1reencoded(RRASAmutant)::RFP; cb-unc-119(+)]II; knl-1(lt53[knl-1::GFP::tev::loxP::3xFlag]) III; Pnphp-4::mNeonGreen-his-72:tbb-2_3'UTR;;gpd-2/3 operon linker-mKate2-PH:unc-54_3'UTR]V	This study	DKC84	**3 & S3**
ltSi1016[pDC337; Pdyf-7::vhhGFP4::ZIF-1::dyf-7_3'UTR; cb-unc-119(+)]#2 I; ltSi48[pOD1038/pJE169; Pknl-1::KNL-1reencoded(MELT repeats mutant)::RFP; cb-unc-119(+)]II]; knl-1(lt53[knl-1::GFP::tev::loxP::3xFlag]) III	This study	DKC99	**3 & S3**
ltSi1016[pDC337; Pdyf-7::vhhGFP4::ZIF-1::dyf-7_3'UTR; cb-unc-119(+)]#2 I]; [Pknl-1::KNL-1 MELT deletion::RFP; cb-unc-119(+)]II; knl-1(lt53[knl-1::GFP::tev::loxP::3xFlag]) III; Pnphp-4::mNeonGreen-his-72:tbb-2_3'UTR;;gpd-2/3 operon linker-mKate2-PH:unc-54_3'UTR]V	This study	DKC105	**3 & S3**

ltSi1038 [pDC344; Pgcy-5::mKate-2::PH_unc-54_3'UTR; cb-unc-119(+)]II; unc-119(ed3)III ?; ptrn-1(lt1::cb-unc-119+)X	This study	DKC112	**1 & S1**
ltSi1016[pDC337; Pdyf-7::vhhGFP4::ZIF-1::dyf-7_3'UTR; cb-unc-119(+)]I; gip-2(lt19[gip-2::GFP]::loxP::cb-unc-119(+)::loxP)I; ltSi1019[pDC353; Pdyf-7::vhhGFP4::ZIF-1::dyf-7_3'UTR; cb-unc-119(+)]II ; unc-119(ed3)III ?; ltSi1055[oxTi365;[pDC344; Pgcy-5::mKate-2::PH_unc-54_3'UTR; cb-unc-119(+)V	This study	DKC129	**S1**
ndc-80(tm5271)/ IV; ltSi1016[pDC337; Pdyf-7::vhhGFP4::ZIF-1::dyf-7_3'UTR; cb-unc-119(+)]I; ltSi122[pDC178;Pndc-80:NDC-80 (8,18,44,51AAAA) reencoded; cb-unc-119(+)]II #1; unc-119(ed3)III?; ltSi1174[oxTi365; pDC591; Pnphp-4::mNeonGreen-his-72:tbb-2_3'UTR;;gpd-2/3 operon linker-mKate2-PH:unc-54_3'UTR]V	This study	DKC160	**3**
ltSi1038 [pDC344; Pgcy-5::mKate-2::PH_unc-54_3'UTR; cb-unc-119(+)]II; unc-119(ed3)III?]; noca-1(ok3692)V/nT1[qIs51](IV;V)	This study	DKC162	**S1**
ndc-80(dha60; NDC-80 deltaTail)IV	This study	DKC400	**5 & S5**
ltSi220[pOD1249/pSW077; Pmex-5::GFP-tbb-2-operon-linker-mCherry-his-11; cb-unc-119(+)]I; ndc-80(dha60; NDC-80 deltaTail)IV	This study	DKC402	**S5**
ltSi1172[pDC591; Pnphp-4::mNeonGreen-his-72:tbb-2_3'UTR;;gpd-2/3 operon linker-mKate2-PH:unc-54_3'UTR]II; unc-119(ed3)III; sax-7(nj48) IV	This study	DKC454	**3 & S3**
unc-119(ed3)III; sax-7(nj48) IV; ltSi1055[oxTi365;[pDC344; Pgcy-5::mKate-2::PH_unc-54_3'UTR; cb-unc-119(+)]]V	This study	DKC559	**1 & S1**
knl-1(dha121(AID::darkg>f>p::::KNL-1))III	This study	DKC614	**4 & S4**
knl-1(dha130; HA::7XGFP11::knl-1)III	This study	DKC693	**2 & 5 & S2**
dhaSi115[pDC1034; hlh-16::splitGFP1-10::rab-3_3'UTR ;;gpd-2 3operon::mSca-I-PH::unc-54_3'UTR; cb-unc-119(+)]II; knl-1(dha130; HA::7XGFP11::knl-1)III	This study	DKC896	**2 & 5 & S2**
dhaSi114[pDC717; lim-4 pro::mNG::PH::unc-54_3'UTR]II; dhaSi73[pDC725; ceh-17 pro::mSca-PH::unc-54_3'UTR; cb-unc-119(+)] IV; unc-119(ed3)III	This study	DKC1156	**5 & S5**
ltSi1016[pDC337; Pdyf-7::vhhGFP4::ZIF-1::dyf-7_3'UTR; cb-unc-119(+)]I #2 ; unc-119(ed3)III?; dhaSi148[pDC897;Pmir-124::mNG-PH::unc-54 3'UTR]II; knl-1(dha121(AID::darkg>f>p::::KNL-1))III; unc-119(ed3)III; ltSi1055[oxTi365;[pDC344; Pgcy-5::mKate-2::PH_unc-54_3'UTR; cb-unc-119(+)]]V	This study	DKC1185	**2 & S2**

ltSi1016[pDC337; Pdyf-7::vhhGFP4::ZIF-1::dyf-7_3'UTR; cb-unc-119(+)]I #2 ; dhaSi148[pDC897;Pmir-124::mNG-PH::unc-54 3'UTR]II; unc-119(ed3)III; ltSi1055[oxTi365;[pDC344; Pgcy-5::mKate-2::PH_unc-54_3'UTR; cb-unc-119(+)]]V	This study	DKC1188	**2 & S2**
dhaSi114[pDC717; lim-4 pro::mNG::PH::unc-54_3'UTR]II; unc-119(ed3)III; dhaSi73[pDC725; ceh-17 pro::mSca-PH::unc-54_3'UTR; cb-unc-119(+)] IV; dhaSi114[pDC717; lim-4 pro::mNG::PH::unc-54_3'UTR]II; ndc-80(dha60; NDC-80 deltaTail)IV	This study	DKC1194	**5 & S5**
ltSi1016[pDC337; Pdyf-7::vhhGFP4::ZIF-1::dyf-7_3'UTR; cb-unc-119(+)]I #2 ; dhaSi305[pDC716; Pgcy-5::mNG::ebp-2::tbb-2_3'UTR::rlaop::mScaPH::unc-54_3'UTR; cb-unc-119(+)]II; unc-119(ed3)III?; knl-1(dha121(AID::darkg>f>p::::KNL-1))III	This study	DKC1211	**4 & S4**
ltSi1016[pDC337; Pdyf-7::vhhGFP4::ZIF-1::dyf-7_3'UTR; cb-unc-119(+)]I #2 dhaSi305[pDC716; Pgcy-5::mNG::ebp-2::tbb-2_3'UTR::rlaop::mScaPH::unc-54_3'UTR; cb-unc-119(+)]II;; unc-119(ed3)III?	This study	DKC1213	**4 & S4**
dhaSi115[pDC1034; hlh-16::splitGFP1-10::rab-3_3'UTR ::gpd-2/3operon::mSca-I-PH::unc-54_3'UTR; cb-unc-119(+)]II; ndc-80[lt126 (ndc-80::7XGFP-11)] IV	This study	DKC1234	**5 & S2**
ltSi1016[pDC337; Pdyf-7::vhhGFP4::ZIF-1::dyf-7_3'UTR; cb-unc-119(+)]I; gip-2(lt19[gip-2::GFP]::loxP::cb-unc-119(+)::loxP)I; ltSi1191[pDC603; Pgcy-5::mNeonGreen-PH::tbb-3'UTR; Pgcy-5::mKate2-snb-1::snb-1_3'UTR; cb-unc-119(+)] II; unc-119(ed3)III?; gip-1(wow3[gfp::gip-1])III	This study	DKC1321	**S4**

Table S2: Sequences of regulatory elements

Promoter	Length	5' end	3' end
P*gcy-5*	3355bp	gtgtcatcaaattccgagcatgtaaaaaaa	ttttcgaaaaattacttattctgatgaaaa
P*dyf-7*	3324bp	ttcatatactttatgtacggcgtacacgca	ctgtttctatttcagatttaaacttcaagt
P*cnd-1*	3230bp	cagctatgacacgtggctctagtaatactt	cttccctgtcatccagttatattttctaca
P*hlh-16*	514bp	ctggaacatcagaaatttgagacttcaaaa	ttcaatatgccgacagcttcatctccgatt
P*mir-124*	2063bp	cgttagattgcttcttctttatgaatttcg	tttatttcttcttcaagtgctctcttctcc
P*ceh-17*	1597bp	gcccgggctacgtaatacgactcacttaag	ccagagggattcaagtgggagcgtgtcatg
P*lim-4*	4605bp	cccatgcagttcaaatactgtcttaaaacc	aacgttttactgtaaaggatgttatttccg
P*mex-5*	488bp	aaatatcagtttttaaaaaattaaaccata	cgataatcaattgaatgtttcagacagaga

Table S3: CRISPR-Cas9 loci & sgRNA sequences used for strain generation

Strain name	Genotype	sgRNA sequence
DKC400	*ndc-80(dha60; NDC-80 deltaTail)IV*	sgRNA1:ggagatcgaagaaaaacggg
		sgRNA2:accaatgatttcacatctcg
DKC614	*knl-1(dha121(AID::darkg>f>p::::KNL-1))III*	sgRNA1:catatttacagccatgtcga
		sgRNA2:cttacgaggctccatcgaca
DKC693	*knl-1(dha130; HA::7XGFP11::knl-1)III*	sgRNA1:catatttacagccatgtcga
		sgRNA2:cttacgaggctccatcgaca

REFERENCES:

Moyle, Mark W., Kristopher M. Barnes, Manik Kuchroo, Alex Gonopolskiy, Leighton H. Duncan, Titas Sengupta, Lin Shao, et al. 2021. "Structural and Developmental Principles of Neuropil Assembly in C. Elegans." *Nature* 591 (7848): 99–104.

Oegema, K., A. Desai, S. Rybina, M. Kirkham, and A. A. Hyman. 2001. "Functional Analysis of Kinetochore Assembly in Caenorhabditis Elegans." *The Journal of Cell Biology* 153 (6): 1209–26.

Rapti, Georgia, Chang Li, Alan Shan, Yun Lu, and Shai Shaham. 2017. "Glia Initiate Brain Assembly through Noncanonical Chimaerin-Furin Axon Guidance in C. Elegans." *Nature Neuroscience* 20 (10): 1350–60.
